# *N*′-Benzyl­idene-2-chloro-*N*-(4-chloro­phen­yl)acetohydrazide

**DOI:** 10.1107/S1600536813032790

**Published:** 2013-12-11

**Authors:** N. Vinutha, S. Madan Kumar, P. C. Shyma, B. Kalluraya, N. K. Lokanath, D. Revannasiddaiah

**Affiliations:** a Department of Studies in Physics, University of Mysore, Manasagangotri, Mysore 570 006, India; b Department of Studies in Chemistry, Mangalore University, Mangalagangotri, Mangalore 574 199, India

## Abstract

In the title compound, C_15_H_12_Cl_2_N_2_O, the atoms not making up the chloro­benzene ring are approximately coplanar (r.m.s. deviation = 0.073 Å). The dihedral angle between these 13 atoms and the chloro­benzene ring is 67.37 (10)°. The C=O and C*sp* ^2^—Cl groups are almost eclipsed [Cl—C—C=O = −6.5 (3)°]. In the crystal, *C*(6) chains linked by C—H⋯O hydrogen bonds result in [100] chains.

## Related literature   

For background to Schiff bases, see: Nithinchandra *et al.* (2013 ▶); Shyma *et al.* (2013 ▶).
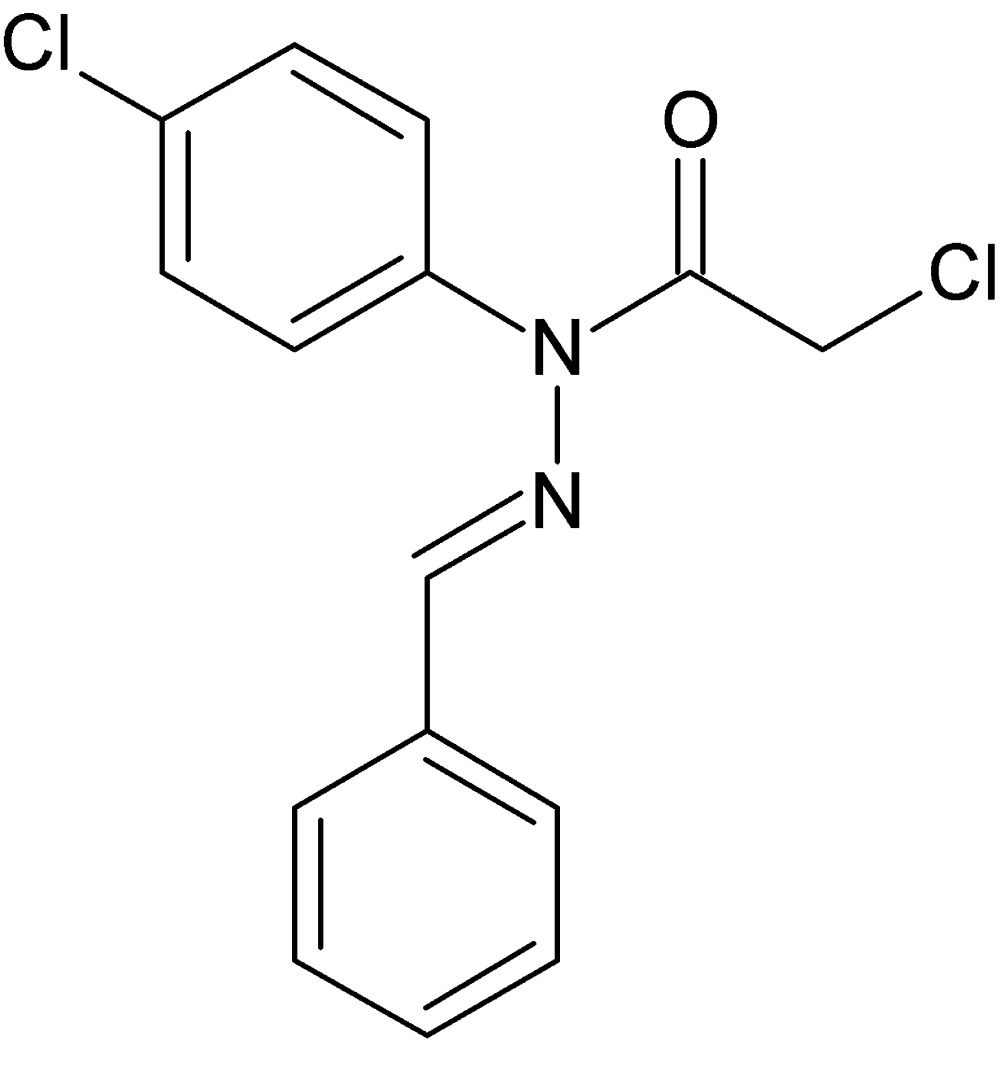


## Experimental   

### 

#### Crystal data  

C_15_H_12_Cl_2_N_2_O
*M* *_r_* = 307.17
Monoclinic, 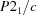

*a* = 5.8548 (5) Å
*b* = 8.8892 (7) Å
*c* = 28.273 (2) Å
β = 93.574 (4)°
*V* = 1468.6 (2) Å^3^
*Z* = 4
Cu *K*α radiation
μ = 3.95 mm^−1^
*T* = 296 K
0.24 × 0.23 × 0.23 mm

#### Data collection  

Bruker X8 Proteum diffractometer
Absorption correction: multi-scan (*SADABS*; Bruker, 2013 ▶) *T*_min_ = 0.451, *T*_max_ = 0.464
10066 measured reflections
2424 independent reflections
2059 reflections with *I* > 2σ(*I*)
*R*_int_ = 0.061

#### Refinement  

*R*[*F* ^2^ > 2σ(*F* ^2^)] = 0.053
*wR*(*F* ^2^) = 0.145
*S* = 1.04
2424 reflections
182 parameters
H-atom parameters constrained
Δρ_max_ = 0.46 e Å^−3^
Δρ_min_ = −0.45 e Å^−3^

### 

Data collection: *APEX2* (Bruker, 2013 ▶); cell refinement: *SAINT* (Bruker, 2013 ▶); data reduction: *SAINT*; program(s) used to solve structure: *SHELXS97* (Sheldrick, 2008 ▶); program(s) used to refine structure: *SHELXL97* (Sheldrick, 2008 ▶); molecular graphics: *Mercury* (Macrae *et al.*, 2008 ▶); software used to prepare material for publication: *PLATON* (Spek, 2009 ▶).

## Supplementary Material

Crystal structure: contains datablock(s) global, I. DOI: 10.1107/S1600536813032790/hb7168sup1.cif

Structure factors: contains datablock(s) I. DOI: 10.1107/S1600536813032790/hb7168Isup2.hkl

Supporting information file. DOI: 10.1107/S1600536813032790/hb7168Isup3.cml

Additional supporting information:  crystallographic information; 3D view; checkCIF report

## Figures and Tables

**Table 1:** Hydrogen-bond geometry (Å, °)

*D*—H⋯*A*	*D*—H	H⋯*A*	*D*⋯*A*	*D*—H⋯*A*
C3—H3⋯O1^i^	0.93	2.40	3.256 (3)	154

Symmetry code: (i) 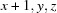.

**Fig. 1.**
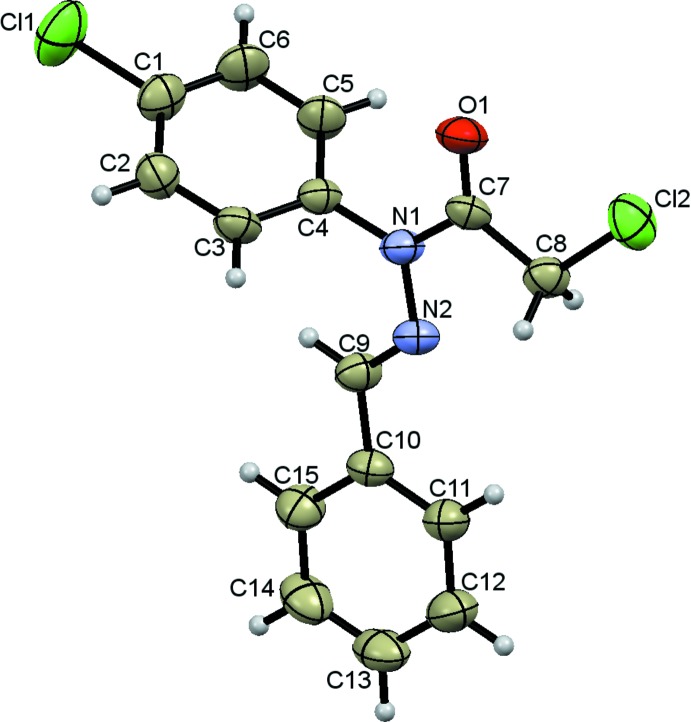
ORTEP diagram of the title compound with 50% probability ellipsoids.

## supplementary crystallographic information

### 1. Comment

Schiff bases are important class of compounds in medicinal chemistry, showing e.g.: antimicrobal (Shyma *et al.*, 2013), and anticonvulsant (Nithinchandra *et al.*, 2013) activities. As part of our studies in this area, we now describe the structure of the title compound, (I), (Fig. 1).

The dihedral angle between the benzene ring is 63.18° (13) and the molecules are linked to one another with hydrogen bonds C3—H3···O1 (Table 1, Fig. 2) along *a*-axis.

### 2. Experimental

4-Chlorophenylhydrazine (0.01 mol) was stirred with benzaldehyde (0.01 mol) in methanol (10 mL) in presence of few drops of acetic acid. The resulting precipitate was filtered and washed with chilled methanol and dried. The resulting Schiff base, 1-benzylidene-2-(4-chlorophenyl)hydrazine (0.01 mol) and triethylamine (0.01 mol) was taken in dioxane solvent. Chloroacetylchloride (0.01 mol) was added to the above mixture at 0–5°C. After completion of the reaction, the mixture was poured on to ice cold water. The precipitated solid was filtered, washed with water and recrystallized from ethanol. Brown blocks were obtained from a 1:2 mixture of DMF and ethanol solution by slow evaporation.

### 3. Refinement

All the H atoms were fixed geometrically (C—H= 0.93–0.96 Å) and allowed to ride on their parent atoms with *U*_iso_(H) =1.5*U*_eq_(*C*-methyl) and = 1.2*U*_eq_(C) for other H atoms.

### Crystal data

**Table:**

C_15_H_12_Cl_2_N_2_O	*F*(000) = 632
*M**_r_* = 307.17	*D*_x_ = 1.389 Mg m^−^^3^
Monoclinic, *P*2_1_/*c*	Cu *K*α radiation, λ = 1.54178 Å
Hall symbol: -P 2ybc	Cell parameters from 2424 reflections
*a* = 5.8548 (5) Å	θ = 3.1–64.9°
*b* = 8.8892 (7) Å	µ = 3.95 mm^−^^1^
*c* = 28.273 (2) Å	*T* = 296 K
β = 93.574 (4)°	Block, brown
*V* = 1468.6 (2) Å^3^	0.24 × 0.23 × 0.23 mm
*Z* = 4	

### Data collection

**Table:**

Bruker X8 Proteum diffractometer	2424 independent reflections
Radiation source: Bruker MicroStar microfocus rotating anode	2059 reflections with *I* > 2σ(*I*)
Helios multilayer optics monochromator	*R*_int_ = 0.061
Detector resolution: 10.7 pixels mm^-1^	θ_max_ = 64.9°, θ_min_ = 3.1°
φ and ω scans	*h* = −3→6
Absorption correction: multi-scan (*SADABS*; Bruker, 2013)	*k* = −10→10
*T*_min_ = 0.451, *T*_max_ = 0.464	*l* = −32→32
10066 measured reflections	

### Refinement

**Table:**

Refinement on *F*^2^	Secondary atom site location: difference Fourier map
Least-squares matrix: full	Hydrogen site location: inferred from neighbouring sites
*R*[*F*^2^ > 2σ(*F*^2^)] = 0.053	H-atom parameters constrained
*wR*(*F*^2^) = 0.145	*w* = 1/[σ^2^(*F*_o_^2^) + (0.0867*P*)^2^ + 0.4858*P*] where *P* = (*F*_o_^2^ + 2*F*_c_^2^)/3
*S* = 1.04	(Δ/σ)_max_ < 0.001
2424 reflections	Δρ_max_ = 0.46 e Å^−^^3^
182 parameters	Δρ_min_ = −0.45 e Å^−^^3^
0 restraints	Extinction correction: *SHELXL97* (Sheldrick, 2008), FC^*^=KFC[1+0.001XFC^2^Λ^3^/SIN(2Θ)]^-1/4^
Primary atom site location: structure-invariant direct methods	Extinction coefficient: 0.0091 (10)

### Special details

**Table:**

Geometry. Bond distances, angles *etc*. have been calculated using the rounded fractional coordinates. All su's are estimated from the variances of the (full) variance-covariance matrix. The cell e.s.d.'s are taken into account in the estimation of distances, angles and torsion angles
Refinement. Refinement on *F*^2^ for ALL reflections except those flagged by the user for potential systematic errors. Weighted *R*-factors *wR* and all goodnesses of fit *S* are based on *F*^2^, conventional *R*-factors *R* are based on *F*, with *F* set to zero for negative *F*^2^. The observed criterion of *F*^2^ > σ(*F*^2^) is used only for calculating -*R*-factor-obs *etc*. and is not relevant to the choice of reflections for refinement. *R*-factors based on *F*^2^ are statistically about twice as large as those based on *F*, and *R*-factors based on ALL data will be even larger.

### Fractional atomic coordinates and isotropic or equivalent isotropic displacement parameters (Å^2^)

**Table:**

	*x*	*y*	*z*	*U*_iso_*/*U*_eq_	
Cl1	0.88967 (18)	1.37204 (11)	0.24673 (3)	0.0826 (4)	
Cl2	0.20082 (12)	0.83970 (9)	−0.00451 (2)	0.0605 (3)	
O1	0.3525 (3)	1.0325 (2)	0.07342 (7)	0.0507 (6)	
N1	0.6637 (3)	0.9050 (2)	0.10193 (7)	0.0379 (6)	
N2	0.8010 (3)	0.7816 (2)	0.09360 (7)	0.0374 (6)	
C1	0.8254 (5)	1.2311 (3)	0.20500 (9)	0.0492 (9)	
C2	0.9644 (5)	1.2137 (3)	0.16799 (9)	0.0474 (8)	
C3	0.9104 (4)	1.1056 (3)	0.13393 (9)	0.0428 (8)	
C4	0.7207 (4)	1.0156 (3)	0.13769 (8)	0.0365 (7)	
C5	0.5846 (5)	1.0340 (3)	0.17556 (9)	0.0483 (8)	
C6	0.6354 (5)	1.1429 (3)	0.20918 (10)	0.0563 (10)	
C7	0.4802 (4)	0.9266 (3)	0.06984 (8)	0.0372 (7)	
C8	0.4559 (4)	0.8111 (3)	0.03078 (9)	0.0433 (8)	
C9	0.9704 (4)	0.7535 (3)	0.12257 (8)	0.0419 (8)	
C10	1.1217 (4)	0.6259 (3)	0.11428 (9)	0.0394 (7)	
C11	1.0788 (5)	0.5252 (3)	0.07751 (9)	0.0472 (8)	
C12	1.2319 (5)	0.4110 (3)	0.07002 (12)	0.0588 (10)	
C13	1.4303 (5)	0.3975 (3)	0.09870 (13)	0.0617 (10)	
C14	1.4735 (5)	0.4958 (3)	0.13506 (12)	0.0611 (10)	
C15	1.3196 (5)	0.6093 (3)	0.14347 (10)	0.0501 (9)	
H2	1.09330	1.27390	0.16590	0.0570*	
H3	1.00200	1.09370	0.10850	0.0510*	
H5	0.45790	0.97230	0.17830	0.0580*	
H6	0.54240	1.15640	0.23430	0.0680*	
H8A	0.45510	0.71110	0.04450	0.0520*	
H8B	0.58570	0.81810	0.01120	0.0520*	
H9	0.99860	0.81410	0.14910	0.0500*	
H11	0.94640	0.53450	0.05780	0.0570*	
H12	1.20130	0.34280	0.04550	0.0710*	
H13	1.53430	0.32140	0.09320	0.0740*	
H14	1.60730	0.48650	0.15440	0.0730*	
H15	1.34880	0.67480	0.16880	0.0600*	

### Atomic displacement parameters (Å^2^)

**Table:**

	*U*^11^	*U*^22^	*U*^33^	*U*^12^	*U*^13^	*U*^23^
Cl1	0.1113 (8)	0.0867 (6)	0.0492 (5)	−0.0170 (5)	0.0013 (4)	−0.0269 (4)
Cl2	0.0502 (5)	0.0824 (6)	0.0476 (4)	−0.0035 (3)	−0.0079 (3)	0.0018 (3)
O1	0.0393 (10)	0.0575 (11)	0.0555 (11)	0.0166 (9)	0.0053 (8)	−0.0058 (8)
N1	0.0360 (11)	0.0419 (11)	0.0361 (10)	0.0096 (9)	0.0041 (8)	−0.0049 (8)
N2	0.0371 (11)	0.0385 (10)	0.0374 (10)	0.0085 (9)	0.0078 (8)	−0.0007 (8)
C1	0.0585 (17)	0.0553 (15)	0.0333 (12)	0.0032 (13)	−0.0010 (11)	−0.0035 (11)
C2	0.0422 (15)	0.0547 (15)	0.0451 (14)	−0.0037 (12)	0.0022 (11)	−0.0007 (11)
C3	0.0374 (14)	0.0517 (14)	0.0406 (13)	0.0050 (11)	0.0124 (10)	−0.0020 (10)
C4	0.0366 (13)	0.0412 (12)	0.0320 (11)	0.0071 (10)	0.0049 (9)	−0.0017 (9)
C5	0.0489 (15)	0.0581 (16)	0.0395 (13)	−0.0045 (12)	0.0150 (11)	−0.0031 (11)
C6	0.0636 (19)	0.0707 (18)	0.0363 (13)	−0.0025 (15)	0.0164 (12)	−0.0100 (12)
C7	0.0316 (13)	0.0444 (13)	0.0366 (12)	0.0043 (11)	0.0109 (9)	0.0023 (10)
C8	0.0421 (14)	0.0516 (14)	0.0366 (12)	0.0030 (11)	0.0061 (10)	−0.0022 (10)
C9	0.0455 (14)	0.0452 (13)	0.0352 (12)	0.0088 (11)	0.0033 (10)	−0.0027 (10)
C10	0.0369 (13)	0.0397 (12)	0.0420 (13)	0.0076 (10)	0.0060 (10)	0.0035 (10)
C11	0.0434 (15)	0.0474 (14)	0.0510 (15)	0.0059 (12)	0.0035 (11)	−0.0056 (11)
C12	0.0563 (18)	0.0488 (15)	0.0724 (19)	0.0063 (13)	0.0139 (15)	−0.0121 (13)
C13	0.0497 (18)	0.0458 (15)	0.091 (2)	0.0124 (13)	0.0147 (16)	0.0043 (15)
C14	0.0450 (16)	0.0559 (16)	0.081 (2)	0.0102 (14)	−0.0062 (14)	0.0127 (15)
C15	0.0516 (17)	0.0460 (14)	0.0519 (15)	0.0089 (12)	−0.0042 (12)	0.0031 (11)

### Geometric parameters (Å, º)

**Table:**

Cl1—C1	1.746 (3)	C11—C12	1.379 (4)
Cl2—C8	1.762 (3)	C12—C13	1.380 (4)
O1—C7	1.210 (3)	C13—C14	1.361 (5)
N1—N2	1.389 (3)	C14—C15	1.383 (4)
N1—C4	1.435 (3)	C2—H2	0.9300
N1—C7	1.376 (3)	C3—H3	0.9300
N2—C9	1.271 (3)	C5—H5	0.9300
C1—C2	1.374 (4)	C6—H6	0.9300
C1—C6	1.372 (4)	C8—H8A	0.9700
C2—C3	1.383 (4)	C8—H8B	0.9700
C3—C4	1.378 (3)	C9—H9	0.9300
C4—C5	1.384 (4)	C11—H11	0.9300
C5—C6	1.376 (4)	C12—H12	0.9300
C7—C8	1.508 (4)	C13—H13	0.9300
C9—C10	1.467 (4)	C14—H14	0.9300
C10—C11	1.383 (4)	C15—H15	0.9300
C10—C15	1.388 (4)		
			
N2—N1—C4	123.31 (18)	C10—C15—C14	120.3 (3)
N2—N1—C7	115.78 (19)	C1—C2—H2	120.00
C4—N1—C7	120.46 (19)	C3—C2—H2	120.00
N1—N2—C9	118.9 (2)	C2—C3—H3	120.00
Cl1—C1—C2	118.9 (2)	C4—C3—H3	120.00
Cl1—C1—C6	119.6 (2)	C4—C5—H5	120.00
C2—C1—C6	121.6 (3)	C6—C5—H5	120.00
C1—C2—C3	119.2 (3)	C1—C6—H6	121.00
C2—C3—C4	120.0 (2)	C5—C6—H6	120.00
N1—C4—C3	119.8 (2)	Cl2—C8—H8A	109.00
N1—C4—C5	120.4 (2)	Cl2—C8—H8B	110.00
C3—C4—C5	119.8 (2)	C7—C8—H8A	109.00
C4—C5—C6	120.5 (3)	C7—C8—H8B	109.00
C1—C6—C5	118.9 (3)	H8A—C8—H8B	108.00
O1—C7—N1	121.0 (2)	N2—C9—H9	120.00
O1—C7—C8	124.1 (2)	C10—C9—H9	120.00
N1—C7—C8	115.0 (2)	C10—C11—H11	120.00
Cl2—C8—C7	110.76 (17)	C12—C11—H11	120.00
N2—C9—C10	120.2 (2)	C11—C12—H12	120.00
C9—C10—C11	122.5 (2)	C13—C12—H12	120.00
C9—C10—C15	118.5 (2)	C12—C13—H13	120.00
C11—C10—C15	118.9 (2)	C14—C13—H13	120.00
C10—C11—C12	120.2 (3)	C13—C14—H14	120.00
C11—C12—C13	120.3 (3)	C15—C14—H14	120.00
C12—C13—C14	119.9 (3)	C10—C15—H15	120.00
C13—C14—C15	120.4 (3)	C14—C15—H15	120.00
			
C4—N1—N2—C9	9.3 (3)	C2—C3—C4—C5	0.1 (4)
C7—N1—N2—C9	−178.4 (2)	N1—C4—C5—C6	−178.2 (2)
N2—N1—C4—C3	65.1 (3)	C3—C4—C5—C6	0.9 (4)
N2—N1—C4—C5	−115.8 (3)	C4—C5—C6—C1	−1.2 (4)
C7—N1—C4—C3	−106.8 (3)	O1—C7—C8—Cl2	−6.5 (3)
C7—N1—C4—C5	72.3 (3)	N1—C7—C8—Cl2	174.25 (17)
N2—N1—C7—O1	−178.6 (2)	N2—C9—C10—C11	−5.1 (4)
N2—N1—C7—C8	0.6 (3)	N2—C9—C10—C15	172.5 (2)
C4—N1—C7—O1	−6.1 (3)	C9—C10—C11—C12	177.1 (3)
C4—N1—C7—C8	173.1 (2)	C15—C10—C11—C12	−0.4 (4)
N1—N2—C9—C10	−178.7 (2)	C9—C10—C15—C14	−176.1 (3)
Cl1—C1—C2—C3	−177.9 (2)	C11—C10—C15—C14	1.5 (4)
C6—C1—C2—C3	0.6 (4)	C10—C11—C12—C13	−0.9 (4)
Cl1—C1—C6—C5	178.9 (2)	C11—C12—C13—C14	1.1 (5)
C2—C1—C6—C5	0.4 (4)	C12—C13—C14—C15	0.0 (5)
C1—C2—C3—C4	−0.8 (4)	C13—C14—C15—C10	−1.3 (4)
C2—C3—C4—N1	179.2 (2)		

### Hydrogen-bond geometry (Å, º)

**Table:**

*D*—H···*A*	*D*—H	H···*A*	*D*···*A*	*D*—H···*A*
C3—H3···O1^i^	0.93	2.40	3.256 (3)	154

Symmetry code: (i) *x*+1, *y*, *z*.
